# Obstructive voiding symptoms following stereotactic body radiation therapy for prostate cancer

**DOI:** 10.1186/1748-717X-9-163

**Published:** 2014-07-24

**Authors:** W Tristram Arscott, Leonard N Chen, Nathan Wilson, Aditi Bhagat, Joy S Kim, Rudy A Moures, Thomas M Yung, Siyuan Lei, Brian T Collins, Keith Kowalczyk, Simeng Suy, Anatoly Dritschilo, John H Lynch, Sean P Collins

**Affiliations:** 1 Department of Radiation Medicine, Georgetown University Hospital, 3800 Reservoir Road, NW, Washington, DC 20007, USA; 2 Department of Urology, Georgetown University Hospital, 3800 Reservoir Road, NW, Washington, DC 20007, USA

**Keywords:** Prostate cancer, SBRT, CyberKnife, IPSS, Retention, Catheterization, TURP

## Abstract

**Background:**

Obstructive voiding symptoms (OVS) are common following prostate cancer treatment with radiation therapy. The risk of urinary retention (UR) following hypofractionated radiotherapy has yet to be fully elucidated. This study sought to evaluate OVS and UR requiring catheterization following SBRT for prostate cancer.

**Methods:**

Patients treated with SBRT for localized prostate cancer from February 2008 to July 2011 at Georgetown University were included in this study. Treatment was delivered using the CyberKnife® with doses of 35 Gy-36.25 Gy in 5 fractions. UR was prospectively scored using the CTCAE v.3. Patient-reported OVS were assessed using the IPSS-obstructive subdomain at baseline and at 1, 3, 6, 9, 12, 18 and 24 months. Associated bother was evaluated via the EPIC-26.

**Results:**

269 patients at a median age of 69 years received SBRT with a median follow-up of 3 years. The mean prostate volume was 39 cc. Prior to treatment, 50.6% of patients reported moderate to severe lower urinary track symptoms per the IPSS and 6.7% felt that weak urine stream and/or incomplete emptying were a moderate to big problem. The 2-year actuarial incidence rates of acute and late UR ≥ grade 2 were 39.5% and 41.4%. Alpha-antagonist utilization rose at one month (58%) and 18 months (48%) post-treatment. However, Grade 3 UR was low with only 4 men (1.5%) requiring catheterization and/or TURP. A mean baseline IPSS-obstructive score of 3.6 significantly increased to 5.0 at 1 month (*p* < 0.0001); however, it returned to baseline in 92.6% within a median time of 3 months. Late increases in OVS were common, but transient. Only 7.1% of patients felt that weak urine stream and/or incomplete emptying was a moderate to big problem at two years post-SBRT (*p* = 0.6854).

**Conclusions:**

SBRT treatment caused an acute increase in OVS which peaked within the first month post-treatment, though acute UR requiring catheterization was rare. OVS returned to baseline in > 90% of patients within a median time of three months. Transient Late increases in OVS were common. However, less than 10% of patients felt that OVS were a moderate to big problem at two years post-SBRT.

## Background

Benign prostatic hyperplasia (BPH) and related lower urinary tract symptoms (LUTS) are a common problem of male aging [1]. LUTS consists of both irritative and obstructive voiding symptoms [1,2]. In men greater than seventy years old, the prevalence of LUTS may be as high as 30% [3]. Older age [4], non-Caucasian race [5], comorbidities [6], and obesity [5] may increase the risk of LUTS.

Obstructive voiding symptoms commonly occur following external beam radiation therapy (EBRT) for prostate cancer and may adversely affect a patient’s quality of life [7,8]. The cause of these symptoms may involve prostatic edema, however the etiology is not fully understood [9-11]. Patients report incomplete emptying, intermittency, weak stream and straining [2], which develop days to weeks after the start of treatment and generally resolve weeks to months following completion of EBRT [12-15]. Patient characteristics including prostatic volume [14,16,17], IPSS score [11,17-19], BPH [20], and prior procedures for BPH [19,21] may contribute to an individual’s risk of radiation-induced UR [22]. Pre-treatment androgen deprivation therapy (ADT) [23,24] and/or post-treatment alpha agonists [25,26] may decrease treatment-related symptoms.

Brachytherapy is an effective and convenient treatment option for clinically localized prostate cancer [27-30]. Obstructive voiding symptoms are the primary urinary morbidity following brachytherapy. Treatment-related factors such as physician experience [31,32], isotope selection [33,34] and/or the number of needle applicators utilized [35,36] may affect the incidence and severity of obstructive voiding symptoms. Acute urinary retention (AUR) is common and may occur in 5-20% of patients [31]. In some cases, prolonged catheterization and/or transurethral resection of the prostate (TURP) are required, which increase the risk of long term urinary incontinence [37]. Intraoperative image-optimized delivery may reduce, but not eliminate, urinary toxicity [38,39]. The negative impact of AUR on quality of life (QoL) is high and lasts for years after the catheter has been removed [40].

Stereotactic Body Radiation Therapy (SBRT) is a safe and effective treatment for clinically localized prostate cancer [41-45]. The larger dose per fraction untilized in SBRT offers the potential radiobiological benefits of hypofractionation [46]. Initial reports suggest that the incidence of urinary obstruction following SBRT is comparable to other external radiotherapy modalities, and may be less than brachytherapy [41-43]. The goal of this study is to report the incidence and prevalence of obstructive voiding symptoms and urinary retention following SBRT for clinically localized prostate cancer.

## Methods

### Patient selection

Georgetown University Hospital established its Prostate SBRT Program in 2006. As of December 2013, 700 prostate cancer patients have been treated with SBRT plus or minus supplemental external beam radiation therapy. At the inception of our program, a prospective database was established to record baseline patient characteristics. At each follow-up visit, toxicity and quality of life data have also been prospectively collected and recorded. Patients eligible for this study were those who had SBRT monotherapy for clinically localized prostate cancer and a minimum of two years of follow-up. Internal Review Board (IRB) approval was obtained for retrospective review of our database.

### SBRT treatment planning and delivery

SBRT treatment planning and delivery were conducted as previously described [47,48]. Briefly, four to six stranded gold fiducials (1013- 2-2, Best Medical International, Inc., Springfield, VA, USA) were placed into the prostate with two to three needle applicators via a trans-rectal or trans-perineal approach. Fused computed tomography (CT) and magnetic resonance magnetic resonance (MR) images were used for treatment planning. The clinical target volume (CTV) included the prostate and the proximal seminal vesicles. The planning target volume (PTV) equaled the CTV expanded 3 mm posteriorly and 5 mm in all other dimensions. The prescription dose was 35-36.25 Gy to the PTV delivered in five fractions of 7-7.25 Gy over one to two weeks. The prescription isodose line was limited to ≥ 75%, which limited the maximum prostatic urethra dose to 133% of the prescription dose. The membranous urethra was contoured and evaluated with dose-volume histogram analysis during treatment planning using Multiplan (Accuray Inc., Sunnyvale, CA). The dose-volume histogram (DVH) goal was for < 50% membranous urethra to receive 37 Gy. To minimize the risk of local recurrence, the dose to the prostatic urethra was not constrained [49]. Prostate position was verified during treatment using paired, orthogonal x-ray images [50].

### Follow-up and statistical analysis

Toxicity and quality of life data were obtained before treatment and during routine follow-up visits every 3 months for the first year and every six months for the second year. Alpha-antagonist utilization was documented at each visit. Physician-reported toxicity was prospectively documented at follow-up visits using the National Cancer Institute (NCI) Common Toxicity Criteria (CTC) version 3.0. Toxicity that occurred between assessments was assigned to the later time point. For example, if a toxicity occurred one week after SBRT it was recorded at one month post-SBRT. Acute toxicity was defined as experiencing toxicity within 6 months of SBRT and late toxicity was defined as occurring greater than 6 months after delivery of SBRT. Grade 1 urinary retention consisted of dribbling or hesitancy not requiring medications for symptom control. Grade 2 urinary retention indicates hesitancy requiring new medication (i.e. alpha-antagonist) or increase in dose of previously prescribed medication. Urinary retention requiring catheterization and/or transurethral resection of the prostate was scored as Grade 3.

Patient-reported obstructive voiding symptoms were assessed via the International Prostate Symptom Score (IPSS), a validated questionnaire where higher scores indicate more severe symptoms [2]. The recall period for the IPSS is one month [51]. The IPSS includes four question related to obstructive symptoms (incomplete emptying, intermittency, weak stream and straining). For each IPSS obstructive question, the responses were grouped into four clinically relevant categories (never, < ½ time, ≥ ½ time and always). The IPSS obstructive subscore (IPSS-O) has been previously defined as the sum of the scores for questions 1, 3, 5, and 6 [52]. Overall IPSS-O scores ranged from 0 - 20. IPSS-0 resolution was defined as a return to within one point of the baseline score [13]. Bother with obstructive urinary symptoms was assessed via Question 4d of the Expanded Prostate Index Composite (EPIC)-26 (“How big a problem, if any, has weak urine stream or incomplete emptying been for you during the last four weeks?”) [53] for which responses were grouped into three clinically relevant categories (no problem, small problem and moderate to big problem).

Student’s *t*-test and Wilcoxon signed-rank test were used to assess differences in ongoing toxicity and quality of life scores in comparison to baseline. Sample medians and ranges were used to describe continuous variables. Actuarial likelihood estimates for late urinary retention ≥ grade 2 and time to IPSS-O resolution were determined using the Kaplan-Meier method. To statistically compare changes between time points, the levels of responses were assigned a score and the significance of the mean changes in the scores was assessed by paired *t* test. The minimally important difference (MID) in IPSS-O score was defined as a change of one-half standard deviation (SD) from the baseline [54]. To limit the effect of attrition bias, statistical analysis was limited to time points in which ≥ 80% of the patient data were available.

## Results

From February 2008 to July 2011, 269 prostate cancer patients were treated per our institutional SBRT monotherapy protocol (Table 1) with a median follow-up of 3 years. They were ethnically diverse with a median age of 69 years (range, 44-90 years). The median prostate volume was 39 cc and 10% had prior procedures for BPH including simple prostatectomy (1 patient), TURP (10 patients), TUNA (1 patient) and TUMT (7 patients).

**Table 1:** Baseline patient characteristics and treatment

		**% Patients (N = 269)**
**Age (y/o)**	Median 69 (44-90)	
	<60	8.2%
	60-69	42.4%
	70-79	41.3%
	≥80	8.2%
**Race**		
	White	55.8%
	Black	37.2%
	Other	7.1%
**Baseline PSA (ng/dL)**	Median 6.2 (0.2-32.5)	
**Prostate Volume (cc)**	Median 39.04 (11.56-138.69)	
**IPSS Baseline**	Median 8 (0-33)	
	Mild (≤7)	49.3%
	Moderate (8-19)	45.5%
	Severe (≥20)	5.2%
**Procedure for BPH prior to RT**		
	Yes	10.0%
	No	90.0%
**α**_**1A **_**Antagonist Utilization**		
	Yes	32.1%
	No	67.9%
**Risk Group**		
	Low	36.8%
	Intermediate	53.2%
	High	10.0%
**Hormonal Therapy**		
	Yes	16.4%
	No	83.6%
**Dose**		
	36.25 Gy	83.3%
	35 Gy	16.4%
	Other	0.4%

The median baseline IPSS was 8, and 32% of patients were using alpha-antagonists prior to SBRT. One patient utilized intermittent catheterization prior to treatment. By D’Amico classification, 99 patients were low-, 143 intermediate-, and 27 high-risk. Sixteen percent of patients received androgen deprivation therapy (ADT) via a gonadotropin-releasing hormone agonist for a median duration of 3 months (range, 3-24 months). Eighty three percent of patients were treated with 36.25 Gy in five 7.25 Gy fractions.

**Figure 1:**
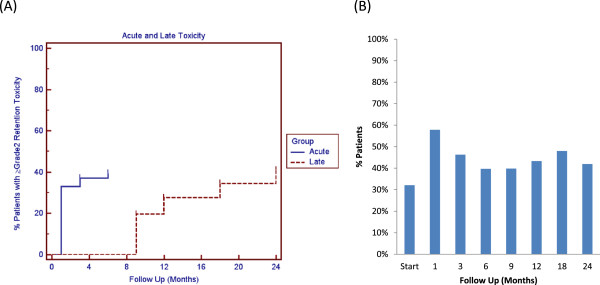
**Acute and late grade 2 urinary retention. (A)** Cumulative acute (≤6 months post-RT) and late (>6 months post-RT) urinary retention ≥ grade 2. **(B)** Proportion of patients utilizing α_1A_ antagonists at each time point.

The prevalence of urinary retention following treatment is shown in Table 2. The corresponding 2-year actuarial incidence of acute and late UR ≥ grade 2 was 39.5% and 41.4%, respectively (Figure 1A). Alpha-antagonist use rose at one month (57.9%) and 18 months (48.0%) post-treatment (Figure 1B). However, Grade 3 UR was low with only 4 men (1.5%) requiring catheterization and/or TURP.

**Table 2:** Prevalence of CTC graded urinary retention at each follow-up

	**1 Mon**	**3 Mon**	**6 Mon**	**9 Mon**	**12 Mon**	**18 Mon**	**24 Mon**
Grade 0	41.7%	57.7%	55.0%	58.7%	56.2%	54.5%	55.4%
Grade 1	21.6%	16.6%	20.7%	19.0%	18.7%	19.3%	21.2%
Grade 2	36.7%	25.7%	23.9%	21.9%	25.1%	25.8%	23.0%
Grade 3	0.0%	0.0%	0.4%	0.4%	0.0%	0.4%	0.5%

**Figure 2:**
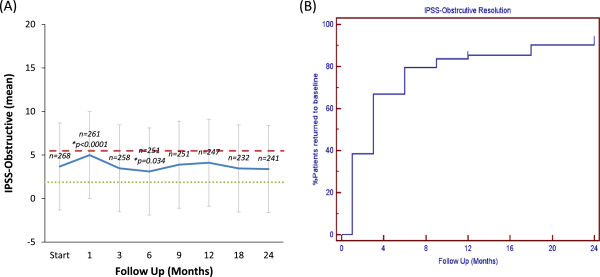
**Obstructive voiding symptoms following SBRT for prostate cancer. (A)** Mean IPSS-O score. The graphs show unadjusted changes in average scores over time. IPSS-O scores range from 0–20 with higher values representing worsening obstructive voiding symptoms. The thresholds for clinically significant changes in scores (½ standard deviation above and below the baseline) are marked with dashed lines. Error bars indicate 95% confidence intervals. **(B)** Time to IPSS-O resolution (return to within one point of the baseline score).

The majority of patients had obstructive voiding symptoms prior to treatment with a mean baseline IPSS-O score of 3.7 (Table 3, Figure 2A). At one month post-SBRT, the mean IPSS-O significantly increased to 5.0 (*p* < 0.0001), but returned to baseline at 3 months (*p* = 0.74, Figure 2A). This increase was of borderline clinical significance (MID = 1.8). The median time to IPSS-O normalization was 3 months (Figure 2B). The IPSS-O returned to baseline in 79.6% of patients by 6 months and 92.6% by 2 years (Figure 2B). Late IPSS-O increases were common, but transient (Figure 2A). Individual obstructive voiding symptoms (incomplete emptying, intermittency, weak stream and straining) followed a similar trend (Table 4).

**Table 3:** Changes in IPSS-O scores following SBRT for prostate cancer

	**Baseline**		**1 Month**		**3 Month**		**6 Month**		**9 Month**		**12 Month**		**18 Month**		**24 Month**	
		**S.D.**	**Change**	**S.D.**	**Change**	**S.D.**	**Change**	**S.D.**	**Change**	**S.D.**	**Change**	**S.D.**	**Change**	**S.D.**	**Change**	**S.D.**
IPSS Obstructive	3.7	3.61	1.3	3.91	−0.2	3.31	−0.6	3.13	0.2	4.06	0.4	0.25	−0.2	3.69	−0.3	3.70

S.D., standard deviation.

**Table 4:** Obstructive voiding symptoms following SBRT for prostate cancer: patient-reported responses to IPSS questions 1 (incomplete emptying), 3 (intermittency), 5 (weak stream) and 6 (straining)

	**Start**	**1 Mon**	**3 Mon**	**6 Mon**	**9 Mon**	**12 Mon**	**18 Mon**	**24 Mon**
**Incomplete Voiding**								
Never	45.1%	26.8%	36.6%	45.6%	40.4%	37.8%	42.9%	44.1%
< 1/2 times	42.2%	52.5%	53.7%	47.2%	45.2%	46.7%	46.0%	43.3%
< 1/2 times	11.2%	18.0%	7.4%	6.8%	12.0%	14.6%	8.8%	8.8%
Always	2.6%	2.7%	2.7%	1.2%	3.2%	1.6%	3.1%	4.2%
*p*		*<0.0001*	*0.747*	*0.1302*	*0.5017*	*0.2546*	*0.5802*	*0.7787*
**Intermittency**								
Never	45.9%	33.3%	45.1%	50.0%	45.2%	43.5%	51.3%	50.8%
< 1/2 times	40.3%	51.0%	47.5%	42.4%	43.6%	39.4%	37.2%	39.1%
< 1/2 times	12.3%	13.4%	5.1%	6.8%	9.2%	15.4%	9.7%	9.2%
Always	1.5%	2.3%	2.3%	0.8%	2.0%	1.6%	1.8%	0.8%
*p*		*0.0025*	*0.1411*	*0.0156*	*0.7464*	*0.8765*	*0.1081*	*0.0225*
**Weak Stream**								
Never	42.7%	26.8%	37.7%	42.8%	37.2%	34.1%	41.2%	40.3%
< 1/2 times	40.8%	46.7%	48.6%	42.0%	43.2%	48.0%	41.6%	45.4%
< 1/2 times	11.2%	22.6%	10.9%	13.2%	16.8%	13.4%	12.8%	10.9%
Always	5.2%	3.8%	2.7%	2.0%	2.8%	4.5%	4.4%	3.4%
*p*		*<0.0001*	*0.9055*	*0.6035*	*0.1365*	*0.1183*	*0.7275*	*0.6782*
**Straining**								
Never	72.4%	60.2%	74.3%	73.9%	68.4%	65.0%	72.4%	75.6%
< 1/2 times	24.6%	33.3%	22.6%	23.7%	25.6%	29.3%	24.4%	20.6%
< 1/2 times	1.9%	5.7%	2.7%	2.4%	4.8%	5.3%	3.1%	3.4%
Always	1.1%	0.8%	0.4%	0.0%	1.2%	0.4%	0.4%	0.4%
*p*		*0.0001*	*0.7224*	*0.2286*	*0.1191*	*0.0482*	*0.7608*	*0.3241*

**Figure 3:**
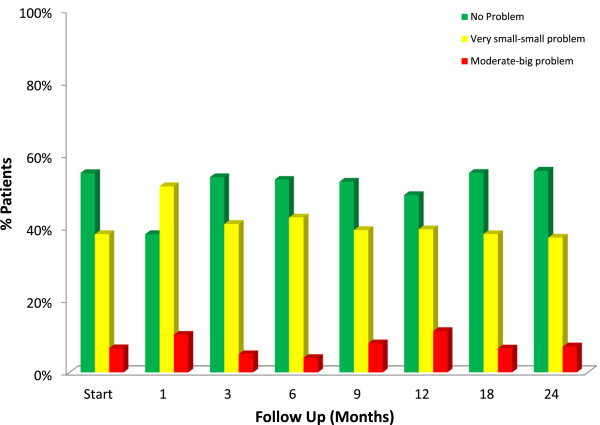
**Bother with weak stream and/or incomplete emptying at baseline and following SBRT for prostate cancer, question 4d of the EPIC-26.** Patients were stratified to three groups: no problem, very small to small problem and moderate to big problem. The percentage of patients in each group at each time point is depicted in the bar chart.

At baseline, 44.9% of our cohort reported some level of bother due to weak stream and/or incomplete emptying with 6.7% of patients feeling it was a moderate to big problem (Table 5, Figure 3). At one month post-SBRT, moderate to big bother with obstructive voiding symptoms increased to 10.4% (*p* < 0.0001), but reduced to 5.1% at 3 months (*p* = 0.79). Although bother declined quickly, a second late transient increase in bother occurred at one year (Table 5, Figure 3). However, only 7.1% of patients felt that weak urine stream and/or incomplete emptying was a moderate to big problem at two years post-SBRT (*p* = 0.6854).

**Table 5:** Bother from weak urine stream and/or incomplete emptying following SBRT for prostate cancer (patient-reported responses to question 4d of the EPIC-26)

	**Start**	**1 Mon**	**3 Mon**	**6 Mon**	**9 Mon**	**12 Mon**	**18 Mon**	**24 Mon**
No Problem	55.1%	38.2%	53.9%	53.2%	52.6%	49.0%	55.1%	55.6%
Very small-small problem	38.2%	51.4%	41.0%	42.8%	39.4%	39.6%	38.2%	37.2%
Moderate-big problem	6.7%	10.4%	5.1%	4.0%	8.0%	11.4%	6.7%	7.1%
N=	267	259	256	250	249	245	225	239
*p*		*<0.0001*	*0.795994*	*0.841946*	*0.197648*	*0.021191*	*1*	*0.68536*

## Discussion

Obstructive voiding symptoms following prostate cancer radiotherapy are common and an important quality of life issue [7]. A better understanding of the pattern of obstructive voiding symptoms following SBRT will enable clinicians to provide more realistic expectations to patients [55]. In this study, we utilized validated QoL questionnaires to comprehensively evaluate obstructive voiding symptoms following SBRT [2,53].

An increase in obstructive voiding symptoms is a common response to prostate radiotherapy. This study shows that SBRT acutely increases all obstructive voiding symptoms (incomplete emptying, intermittency, weak stream and straining) in a similar manner. The 2-year actuarial incidence rates of acute and late UR ≥ grade 2 in this series were 39.5% and 41.4%, respectively. New alpha-antagonist use or alpha-antagonist dose increase were the most common indications of grade 2 toxicities. It is our institutional policy to proscribe alpha-antagonists for mild to moderate LUTS, and this may explain the high rate of GU toxicity ≥ grade 2 seen in this study [47]. Nonetheless, our results appear similar to those reported for IMRT [56] and brachytherapy [31]. To maximize patient comfort, it is currently our institutional policy to initiate prophylactic alpha antagonist use [57] and to treat on an every other day schedule [58].

Post-SBRT urinary symptoms may be exacerbated by high radiation doses to the center of the prostate [47]. With the aim of reducing urinary symptoms, we have modified our institutional protocol. Specifically, we have reduced the anterior/superior PTV expansion to reduce the bladder neck dose. In addition, it is now our practice to prescribe to ≥ 80% isodose line to reduce the central hot spot that may involve the prostatic urethra. We believe that such modifications will reduce the incidence and severity of urinary symptoms.

Due to its effectiveness and convenience, brachytherapy is a common treatment option for prostate cancer. Post-implant UR is a common (5-20%) toxicity that may impact long-term quality of life. Risk factors for post-implant UR include large prostate volume, high pretreatment IPSS score and BPH. Our patients were old with poor baseline urinary function, yet the incidence of UR following SBRT in this series was low (<5%). This low incidence was possibly due to limited needle trauma and/or the use of neoadjuvant ADT in patients with large prostate volumes and high pretreatment IPSS scores [24]. Because of the limited number of events, this study could not determine risk factors for UR following SBRT. If cancer control is similar, SBRT may be a convenient treatment option for patients at high risk of UR following brachytherapy [14,19].

IPSS resolution following brachytherapy varies from months to years [13,15,31]. As seen in other SBRT series [41], our mean IPSS scores returned to baseline within three months post-SBRT. A minority of patients experienced a transient increase in obstructive voiding symptoms greater than six months after the completion of SBRT. As with brachytherapy, late urinary symptom flare [59-61] occurred in a minority of our patients and resolved with conservative management. Knowledge of this late increase in obstructive voiding symptoms and their resolution with conservative management will enable clinicians to address patient concerns and prevent unnecessary catheterization and/or TURP.

Bother is defined as the degree of interference or annoyance caused by a symptom [15,62]. Similar to other radiation modalities, bother with weak stream and/or incomplete voiding plateaued within the first month following treatment with 10% of men reporting a moderate to big problem. This change compares favorably to that reported at two months with conventionally fractionated external beam radiation therapy (23%) and brachytherapy (40%) [7]. As see with external beam radiation therapy, this increase in bother was transient and returned to baseline by 3 months post-SBRT. A second increase in bother occurred 12 months post-SBRT with 11% of patients reporting moderate to big bother at this time point. This change is comparable to that reported at 12 months with intensity modulated radiation therapy (15%) [8], proton therapy (11%) [8] and brachytherapy (18%) [7]. Unlike brachytherapy, though, bother following SBRT returned to near baseline by two years.

Our study has several limitations. There was a poor correlation between physician-reported toxicity and patient-reported outcomes. The prescription of an alpha-antagonist for obstructive urinary symptoms was not guided by a standard protocol [12]. It is possible, that many patients with mild obstructive urinary symptoms received alpha-antagonists and were inappropriately scored a Grade 2 toxicity. Alternatively, the high rate of alpha-antagonist utilization in this study may have hidden the true incidence of patient reported obstructive voiding symptoms with SBRT. In addition, most late obstructive voiding symptoms were transient and associated bother may have been missed due to the timing of questionnaire administration [51].

## Conclusions

SBRT is a convenient treatment option for patients with clinically localized prostate cancer. Treatment resulted in an acute increase in obstructive urinary symptoms which peaked within the first month post-SBRT. These symptoms returned to baseline in the majority of patients by 6 months and in > 90% by 2 years. The risk of acute urinary retention requiring catheterization was low, and late increases in obstructive voiding symptoms were common, though transient. Overall, bother with obstructive voiding symptoms was at baseline two years post SBRT.

## Abbreviations

ADT: Androgen deprivation therapy; AUR: Acute urinary retention; BED: Biologically effective dose; BPH: Benign prostatic hyperplasia; CT: Computed tomography; CTV: Clinical target volume; DVH: Dose-volume histogram; EQD2: Equivalent dose in 2-Gy fractions; EPIC: Expanded Prostate Index Composite; GTV: Gross target volume; Gy: Gray; IMRT: Intensity modulated radiation therapy; IPSS: International Prostate Symptom Score; IRB: Internal Review Board; PTV: Planning target volume; QoL: Quality of life; MID: Minimally important difference; MR: Magnetic resonance; SD: Standard deviation; SBRT: Stereotactic body radiation therapy; UI: Urinary incontinence; UR: Urinary retention.

## Competing interests

SP Collins and BT Collins serve as clinical consultants to Accuray Inc. The Department of Radiation Medicine at Georgetown University Hospital receives a grant from Accuray to support a research coordinator. The other authors declare that they have no competing interests.

## Authors’ contributions

WA and LC are lead authors who participated in manuscript drafting, table/figure creation, and manuscript revision. JW, JP, JK, RM, and TY aided in data collection and table/figure creation. SL is the dosimetrist who contributed dosimetric data and figures. SL, BC, KK, SS, AD, and JL are senior authors who aided in drafting the manuscript and manuscript revision. SC is the corresponding author who initially developed the concept, and drafted and revised the manuscript. All authors read and approved the final manuscript.
